# The Surgical Management of Severe Scoliosis in Immature Patient with a Very Rare Disease Costello Syndrome—Clinical Example and Brief Literature Review

**DOI:** 10.3390/life14060740

**Published:** 2024-06-10

**Authors:** Pawel Grabala, Piotr Kowalski, Marek J. Rudziński, Bartosz Polis, Michal Grabala

**Affiliations:** 1 Department of Pediatric Orthopedic Surgery and Traumatology, University Children’s Hospital, Medical University of Bialystok, Waszyngtona 17, 15-274 Bialystok, Poland; marek.j.rudzinski@gmail.com; 2 Paley European Institute, Al. Rzeczypospolitej 1, 02-972 Warsaw, Poland; 3 Department of Neurosurgery, Medical University of Bialystok, M. Sklodowskiej-Curie 24A, 15-276 Bialystok, Poland; 4 Department of Neurosurgery, Regional Specialized Hospital, ul. Dekerta 1, 66-400 Gorzow Wielkopolski, Poland; pkowal72@gmail.com; 5 Department of Neurosurgery and Neurotraumatology, Polish Mother’s Memorial Hospital Research Institute, 281/289 Rzgowska St., 93-338 Lodz, Poland; jezza@post.pl; 6 2nd Clinical Department of General and Gastroenterogical Surgery, Medical University of Bialystok, ul. M. Skłodowskiej-Curie 24A, 15-276 Bialystok, Poland; michal@grabala.pl

**Keywords:** Costello syndrome, severe scoliosis, pedicle screw, scoliosis, spinal deformity

## Abstract

Background: Costello syndrome (CS) is a rare genetic syndrome in which, due to the occurrence of a mutation in the HRAS gene on chromosome 11 that causes the manifestation, a set of features such as a characteristic appearance, many congenital defects, intellectual disability and a genetic predisposition to cancer, friendly personality, and others can be identified. CS is very rare, with an incidence of ~1/300,000, but it belongs to one of the largest groups of congenital syndromes, called RASopathies, occurring with an incidence of 1/1000 people. Scoliosis and kyphosis, as well as other spinal defects, are common, in 63% and 58% of patients, respectively, and a study conducted among adult patients showed the presence of scoliosis in 75% of patients; there may be excessive lordosis of the lumbar section and inverted curvatures of the spine (lordosis in the thoracic section and kyphosis in the lumbar section). The aim of our study is to present a case report of treatment of severe scoliosis of 130 degrees in a 14-year-old patient with Costello syndrome, with coexisting Chiari II syndrome and syrinx in the absence of skeletal maturity. This patient underwent foramen magnum decompression 3 months before planned surgical correction for severe scoliosis. The patient was qualified for surgical treatment using magnetically controlled growing rods (MCGR). After spine surgery using MCGR, we gradually performed MCGR distraction over the next 2 years; we performed the final surgery, conversion to posterior spinal fusion (PSF) with simultaneous multi-level Ponte osteotomy, which gave a very good and satisfactory surgical result. In the perioperative period, two serious complications occurred: pneumothorax caused by central catheter and gastrointestinal bleeding due to previously undiagnosed gastrointestinal varices. This case shows that the treatment of severe and neglected scoliosis is complicated and requires special preparation and a surgical plan with other cooperating specialists. The scoliosis was corrected from 130 degrees to approximately 48 degrees, sagittal balance was significantly improved, and the surgical outcome was very pleasing, significantly improving quality of life and function for the patient.

## 1. Introduction

Costello syndrome (CS) is a rare genetic syndrome in which, due to the occurrence of a mutation in the HRAS gene on chromosome 11 that causes the manifestation, a set of features such as a characteristic appearance, many congenital defects, intellectual disability and a genetic predisposition to cancer, friendly personality, and others can be identified [1,2,3]. The disease was first described by John M. Costello in 1971 [4]. CS is very rare, with an incidence of ~1/300,000 [5,6], but it belongs to one of the largest groups of congenital syndromes, called RASopathies, occurring with an incidence of 1/1000 people [2,7]. Apart from CS, this group includes, among others, Noonan syndrome and neurofibromatosis type 1 [8]. Costello syndrome is associated with many musculoskeletal disorders, including hypotonia, hypermobility of small joints, ulnar subluxation of the fingers and wrists, joint contractures (primarily of the shoulder and elbow), tight Achilles tendons (which require surgery in almost all patients), and flat feet, as well as hip dysplasia, foot deformities and abnormal chest structure [9,10,11,12,13,14,15]. Patients have a characteristic posture with an anteriorly flexed trunk at the waist. Scoliosis and kyphosis, as well as other spinal defects, are common, in 63% and 58% of patients, respectively [9,10,11,13,14], and a study conducted among adult patients showed the presence of scoliosis in 75% of patients; there may be excessive lordosis of the lumbar section and inverted curvatures of the spine (lordosis in the thoracic section and kyphosis in the lumbar section) [9,10,11,12,13,14]. There are disorders in the structure of muscles and bones which reduce mineral density, which may lead to the development of osteoporosis [11,12,15].

The aim of our study is to present a case report of treatment of severe scoliosis of 130 degrees in a 14-year-old patient with Costello syndrome with coexisting Chiari II syndrome and syrinx in the absence of skeletal maturity.

## 2. Case Presentation

**Figure 1:**
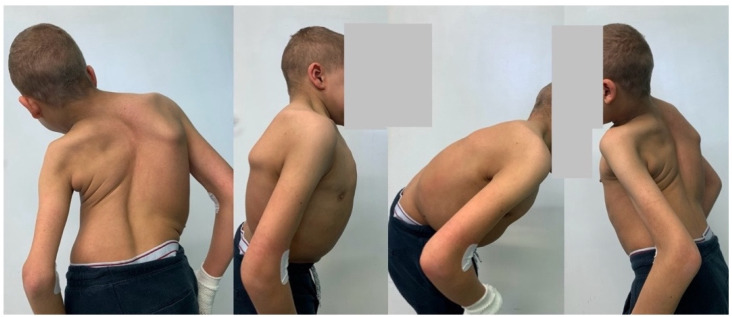
Clinical pictures of 14-year-old immature patient with Costello syndrome, severe spinal deformity measured of 130 degrees.

**Figure 2:**
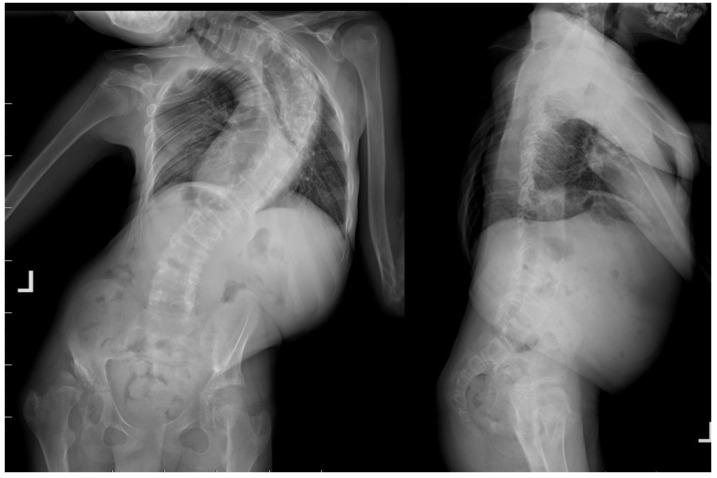
Radiological standing X-rays AP and LAT of 14-year-old immature patient with Costello syndrome, severe spinal deformity measured of 130 degrees.

**Figure 3:**
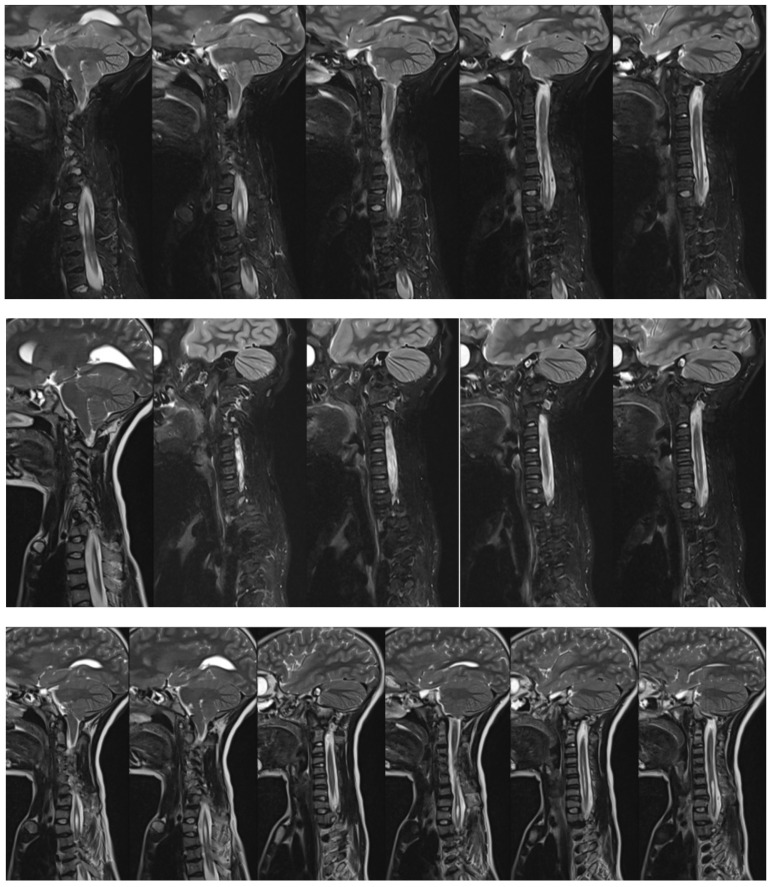
MRI screening of 14-year-old boy with Costello syndrome before surgical treatment of severe scoliosis showed Chiari II and syrinx.

**Figure 4:**
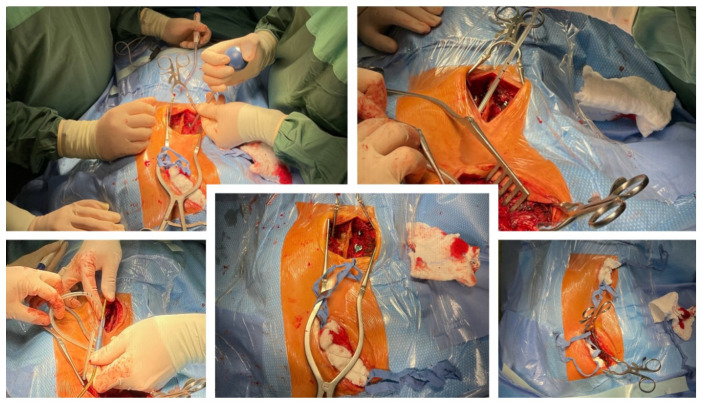
Intraoperative pictures showed implantation of MCGRs via less invasive posterior approach.

**Figure 5:**
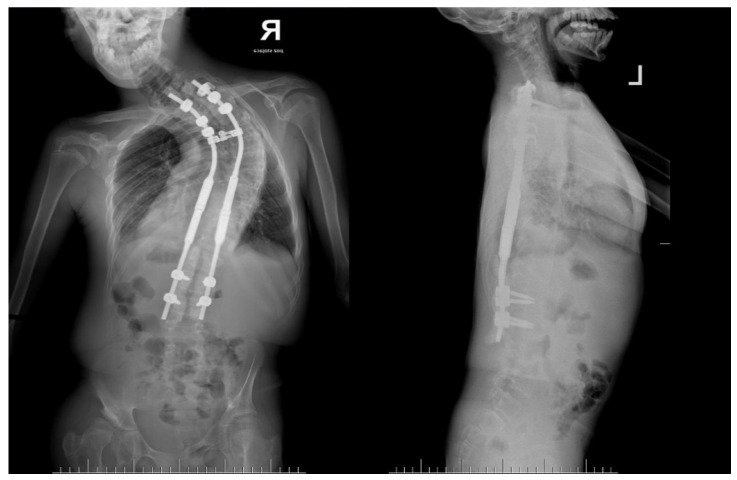
Radiological standing X-rays AP and LAT of 14-year-old boy at 1 year of follow-up showed correction of main curve from 130 degrees to 80 degrees.

**Figure 6:**
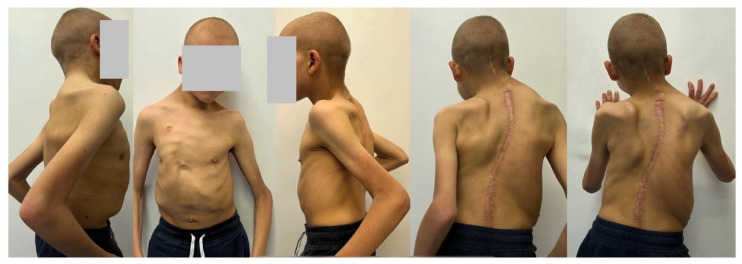
Clinical pictures showed good surgical outcomes of treatment course of the patient with Costello syndrome at 2 years of follow-up after undergoing conversion from MCGR to posterior spinal fusion.

**Figure 7:**
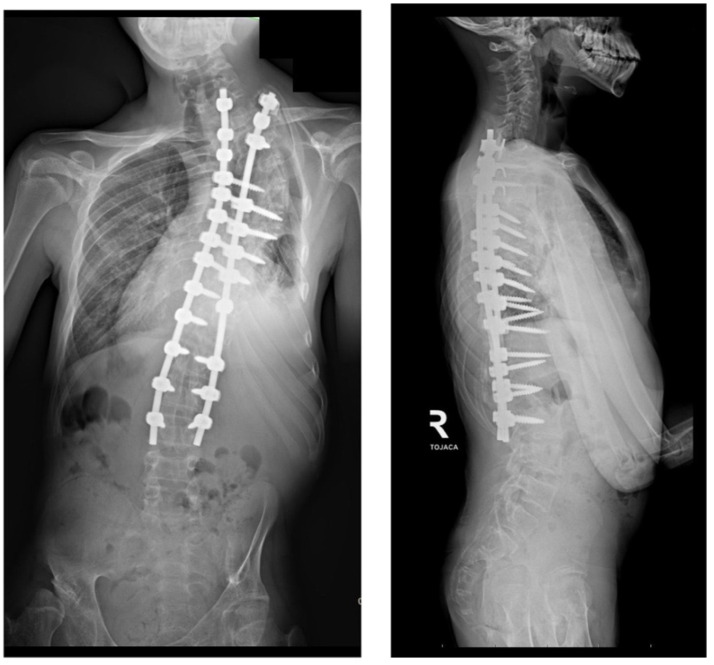
Radiological standing X-rays AP and LAT of the patient with Costello syndrome at 2 years of follow-up after undergoing conversion from MCGR to posterior spinal fusion.

A 14-year-old boy was admitted to our clinic due to rapidly progressing scoliosis. He had previously been treated by other physicians and had an extensive history of conservative treatment of spinal deformities, including the use of a Cheneaux brace. Despite all the efforts made, the scoliosis continued to progress. During a visit to our clinic and examination, we revealed a spine curvature of 130 degrees (Figure 1 and Figure 2). The boy used a wheelchair due to severe walking problems. He was able to walk about 50–100 m on his own, but he developed pain in his spine and the left hip joint, which was dislocated. Although the boy was 14 years old, his mental development could be estimated at the age of a seven-year-old child. Additionally, we diagnosed that both of his feet were deformed, flat-valgus, making walking difficult, and had not been treated so far. Contractures were also revealed in all joints of the upper and lower limbs, slightly limiting mobility. However, the biggest functional problem was severe scoliosis, which was stiff and correctable only in about 30 percent. We recommended tests to prepare for and qualify for spine surgery. Spinal MRI diagnosed Chiari II syndrome and syrinx (Figure 3). This patient underwent foramen magnum decompression 3 months before planned surgical correction for severe scoliosis. Then, due to severe scoliosis of 130 degrees, bone immaturity and insufficient T1–T12 dimensions, the patient was qualified for surgical treatment using magnetically controlled growing rods (MCGR), due to surgical technique described in the literature [16,17,18]. Other less invasive techniques for traction spinal deformities [19,20,21,22], such as halo gravity traction (HGT), could not be used due to the fact that HGT treatment is contraindicated in the presence of spinal pathologies such as syrinx, spinal tumors, increasing the risk of neurological complications during HGT course. Also, instability of the spine in the occipital–cervical and cervical region disqualifies the patient from such treatment. In our case, the patient underwent Chiari II syndrome decompression and had syrinx. For this reason, we considered MCGR treatment to be the least invasive [19,22]. We did not consider anterior release because it is a rather antiquated technique and the superiority of other methods of surgical treatment without disturbing the continuity of the chest has been proven [19,21,23,24,25,26]. This solution was very good for the patient because our surgical technique provided minimal surgical technique, implantation of screws and rods through two small incisions, and correction by distraction under the control of spinal cord neuromonitoring (Figure 4) [23]. In the period after MCGR implantation and subsequent spine surgery, pediatric orthopedists performed two foot deformation correction surgeries (Figure 5). After spine surgery using MCGR, we gradually performed MCGR distraction over the next 2 years, and after no further distractions were possible, we performed the final surgery, conversion to PSF with simultaneous multi-level Ponte osteotomy, which gave a very good and satisfactory surgical result [24]. In the perioperative period, two serious complications occurred: pneumothorax caused by central catheter, and gastrointestinal bleeding due to previously undiagnosed gastrointestinal varices. Pneumothorax was treated with pleural drainage (3 days), and bleeding from the gastrointestinal tract was treated gastroscopically. Finally, the patient was placed in an upright position on the 4th postoperative day and left the hospital on the 8th postoperative day, with full satisfaction with the treatment. The patient was monitored on an outpatient basis every 3 months. After the surgery, his body’s performance improved significantly and his spine pain decreased. T1–T12 height and T1–S1 improved from 156 mm and 268 mm preoperatively, respectively, to 194 mm and 332 mm after MCGR placement, respectively, and 215 mm and 368 mm after definitive surgery and posterior final fusion. He can walk about 2 km a day without any pain (Figure 6 and Figure 7).

## 3. Discussion

This report describes a very rapid progression of spinal deformation in the course of CS, which led to a curvature of 130 degrees, which is very difficult to treat and significantly burdens the patient’s performance. To date, no cases of rapid progression of spinal deformation in the presence of Costello syndrome have been observed in the medical literature. A previous report described the prevalence of scoliosis as 63% and kyphosis as 58% in patients with CS, similar to the values found in these data [9,10,11]. In our opinion, the patient was not properly diagnosed earlier—MRI of the spine was not performed and Chiari II and syrinx were not diagnosed earlier. This could have had a significant impact on the faster progression of the curvature. Our study sheds light on spinal deformities typical of CS. Although it is important to emphasize the speculative nature of these conclusions, which are not based on subsequent data, based on descriptions of other CS in the literature, spinal deformity appears to be static or slowly progressive if it occurs spontaneously, without concomitant spinal cord defects. To prevent the risk of faster progression, MRI of the brain and spine should be performed to rule out structural abnormalities. Moreover, patients with CS-related spinal deformity require prolonged follow-up due to delays in achieving skeletal maturity, as was the case with our patient.

These diseases have their pathogenesis in germline mutations in genes encoding components and regulators of the Ras/MAPK pathway, which plays a very important role in cell cycle control, cell differentiation, growth, and aging. In CS, the mutation affects the HRAS [8] gene and most often occurs de novo. In some cases, the diagnosis may be made already in fetal life, when such abnormalities are visible as an increased amount of amniotic fluid (most often), macrocephaly, macrosomia, shortened long bones and ventriculomegaly, thickened nuchal fold, ulnar subluxation of fingers and wrists, tachyarrhythmias [1,4,27,28]; typical appearance at birth is a characteristic facial appearance with hypertelorism, low-set, posteriorly angulated ears, short nose with bulbous tip, full lips, macrostomia; and hands form clenched fists with an overlap of the fingers [1]. Molecular techniques are used to clearly confirm a clinically suspected diagnosis to detect the pathogenic variant of the HRAS gene. In the first years of life, the most serious problems that affect patients with CS are difficulties in eating and impaired growth resulting from abnormalities in the digestive, nervous, and muscular systems and with increased resting energy expenditure caused by an impairment of the Ras/MAPK [1,29] pathway. Although children are usually born with normal weight and length, body weight decreases quickly to below the population average [30]. Intragastric feeding is usually required for the first 3–4 years of life; then, children gain the ability to feed normally, but reduced height and body weight persist throughout life, leading to a final height in the range of 135-150 cm [28,31]. Growth disorders prompted researchers to create centile charts for patients with CS [27].

Virtually all patients reach developmental milestones with delays, cognitive disorders always occur, and approximately 80% have intellectual disabilities [1,32]. Patients under 4 years of age often meet the classification criteria for autism spectrum disorders, but from approximately 8-10 years of age, they are friendly and outgoing, which is considered one of the characteristic features of this syndrome [1,33,34]. Neurological disorders are caused by anatomical and functional abnormalities. Macrocephaly and macrencephaly, as well as posterior fossa crowding, occur, which may lead to intussusception of the cerebellar tonsils, resulting in the development of Chiari type 1 malformation and other neurological disorders [1]. Most patients with CS have visual problems, such as a lack of stereoscopic vision, refractive disorders, strabismus, nystagmus, optic nerve anomalies and ptosis, which reduce the quality of life and hinder neurodevelopmental skills’ acquisition [1,13,14,15,35].

Cardiovascular diseases occur in 85% of patients and constitute an important factor of co-morbidity and mortality, especially in the first years of life. The most common are hypertrophic cardiomyopathy (in approximately 60% of patients) and congenital heart defects (45%), among which the most common are pulmonary valve stenosis; arrhythmias and supraventricular tachycardias often occur [1,3,6,36]. Structural and functional disorders of the respiratory tract and lung tissue may cause premature birth and increased mortality in the first months of life and may constitute a significant problem in everyday life, and, in combination with very frequent dental and oral findings in CS, may pose difficulties for the anesthesiologist during surgery [1,28,37]. Ectodermal features have no functional effects but contribute to the characteristic phenotype of patients with CS; these are papillomata in the nasal and perianal regions, sparse and curly hair, full, thick eyebrows, deep palmar and plantar creases, pachydermatoglyphia, and loose, soft skin. Cutis laxa, keratoderma, hyperkeratosis and eczema are less common, but they reduce the quality of life of patients and their relatives [6]. Endocrine problems in CS include neonatal hyperinsulinism, hypoglycemia, growth hormone and cortisol deficiency, and pubertal disorders. The increased risk of malignancy in CS is very clinically important. Patients have a more than 40-fold increased risk of developing cancer compared to the general population. Rhabdomyosarcoma and neuroblastoma occur primarily in childhood, and bladder cancer occurs in adolescence and early adulthood [1,6]. Determining the life expectancy of patients with CS requires further research, but many patients who survive to adulthood have been described [6,31]. 

Considering the numerous health problems described above affecting patients with CS, patients should be cared for by many specialists from the beginning of life, and in the case of prenatal diagnosis, starting from fetal life. In recent years, intensive research has been carried out on new therapies that could be used in RASopathies, including CS. Hope is provided primarily by drugs that affect the Ras/MAPK pathway, but they require research [38,39,40,41].

## 4. Conclusions

This case shows that the treatment of severe and neglected scoliosis is complicated and requires special preparation and a surgical plan with other cooperating specialists. To reduce the risk of potential complications, staged treatment should be considered to gradually and safely correct the curved spine, allowing for satisfactory correction of the deformity with excellent healing potential. Scoliosis was corrected from 130 degrees to approximately 48 degrees, sagittal balance was significantly improved, T1–T12 height and T1–S1 improved from 156 mm and 268 mm preoperatively, respectively, to 215 mm and 368 mm after definitive surgery and posterior final fusion. The surgical outcome was very pleasing, significantly improving quality of life and function for the patient. However, the treatment is non-standard and unexpected complications may occur.

## Data Availability

Not applicable.
